# Sample chamber for synchrotron based *in-situ* X-ray diffraction experiments under electric fields and temperatures between 100 K and 1250 K

**DOI:** 10.1107/S1600577520014344

**Published:** 2021-01-01

**Authors:** Melanie Nentwich, Tina Weigel, Carsten Richter, Hartmut Stöcker, Erik Mehner, Sven Jachalke, Dmitri V. Novikov, Matthias Zschornak, Dirk C. Meyer

**Affiliations:** a Institute of Experimental Physics, TU Bergakademie Freiberg, 09596 Freiberg, Germany; b Leibniz-Institut für Kristallzüchtung, 12489 Berlin, Germany; c NaMLab gGmbH, Nöthnitzer Strasse 64a, 01187 Dresden, Germany; d DESY Photon Science, Deutsches Elektronen-Synchrotron DESY, 22607 Hamburg, Germany; e Institute of Ion Beam Physics and Materials Research, Helmholtz-Zentrum Dresden-Rossendorf, 01328 Dresden, Germany

**Keywords:** instrumentation, X-ray diffraction, electric field, sample environment

## Abstract

Many scientific questions require X-ray experiments conducted at varying temperatures, sometimes combined with the application of electric fields. Here, a customized sample chamber developed for beamlines P23 and P24 of PETRA III at DESY to suit these demands is presented. The chamber body consists mainly of standard vacuum parts housing the heater/cooler assembly supplying a temperature range of 100 K to 1250 K and an *xyz* manipulator holding an electric contact needle for electric measurements at both high voltage and low current. The chamber is closed by an exchangeable hemispherical dome offering all degrees of freedom for single-crystal experiments within one hemisphere of solid angle. The currently available dome materials (PC, PS, PEEK polymers) differ in their absorption and scattering characteristics, with PEEK providing the best overall performance. The article further describes heating and cooling capabilities, electric characteristics, and plans for future upgrades of the chamber. Examples of applications are discussed.

## Abstract 2

A custom sample chamber developed for synchrotron X-ray experiments conducted at varying temperatures combined with the application of electric fields is presented. The chamber features an exchangeable hemispherical dome and an *xyz* manipulator for electric contacting of the sample. The article describes heating and cooling capabilities, exemplary experiments and plans for future upgrades of the chamber.

## 1. Introduction   

One of the major advantages of X-rays over other probes in material characterization is the compatibility with complex sample environments based on the large X-ray penetration depth. This ability allows the study of physical properties of materials under real conditions and of structural transitions due to external perturbations by various physical fields, like temperature and electric field. The determination of cross-coupling coefficients of such material properties can be used to study ferroelectricity and related effects, *e.g.* piezo- and pyroelectricity, but also higher-order effects like electro­striction and flexoelectricity. For instance, the knowledge of piezo- and pyroelectric coefficients at non-ambient conditions is essential for the development of high-temperature-stable sensors and actuators. Up to now, commercial sample environments for X-ray diffraction (XRD) experiments providing access to a large solid angle are rare [*e.g.* Anton Paar DCS 500 and DHS 1100 (Anton Paar, 2020*a* ▸,*b* ▸ ▸) or ADC XRD-1500 (Advanced Design Consulting, 2020 ▸)]. In particular, systems combining low or high temperatures with high electric fields and low sample-stage-induced current background in vacuum are not available. However, customized chambers for nearly every purpose can be designed individually (Richard *et al.*, 2017 ▸).

The electrical and simultaneous structural investigation represents the major application of this chamber. For instance, we can determine pyroelectric coefficients using the Sharp–Garn method (Sharp & Garn, 1982 ▸; Garn & Sharp, 1982 ▸) or characterize ferroelectric hysteresis loops (Sawyer & Tower, 1930 ▸), by implementing well defined, alternating temperature and/or electrical functions. In parallel, crystal structure analysis is possible via standard XRD or in combination with resonant techniques like X-ray absorption fine structure, and resonant X-ray diffraction (RXD) (Zschornak *et al.*, 2014 ▸; Richter *et al.*, 2018 ▸) comprising various kinds of scans such as energy, azimuthal, and rocking scans. For instance, a simultaneous structural and electrical characterization of pyroelectrics was discussed recently, where the spontaneous polarization is determined from structure as a function of temperature using a semi-theoretical approach based on Born effective charges from density functional theory calculations (Weigel, 2016 ▸).

The presented sample environment is particularly apt for the study of polar materials (*e.g.* ferroelectrics discussed in Section 8), but also of charge/discharge processes of batteries and their parts at different temperatures for an improved understanding of the underlying processes, which are of particular importance for renewable energy research. The combination of electrical measurements at varying temperatures with (powder) XRD measurements allows the analysis of volume changes caused by migrating and intercalating ions (Yoon *et al.*, 2006 ▸; Weigel *et al.*, 2019 ▸). Simultaneous resonant scattering experiments give insights into changes of the oxidation state and of the local environment (Terada *et al.*, 2000 ▸; Giorgetti *et al.*, 2019 ▸).

Here, we present a highly modular sample chamber for the application of temperature and electric field in large ranges, in combination or individually, while simultaneously characterizing the structure of an extended crystal in non-ambient conditions using synchrotron radiation. The chamber provides X-ray access to a full hemisphere of solid angle and a large angular degree of freedom, allowing, for instance, to measure several oblique reflections without readjustement as described in Section 8.1 This large angular flexibility allows XRD measurements, for example for structure determination and reciprocal space mapping on single-crystalline samples or grazing-incidence diffraction on thin films. The available temperature range spans from 100 K to 1250 K, and the electrical equipment is designed for measuring currents between 1 pA and 1 A at voltages between 1 V and 5 kV.

## 2. Overall specifications   

**Figure 1:**
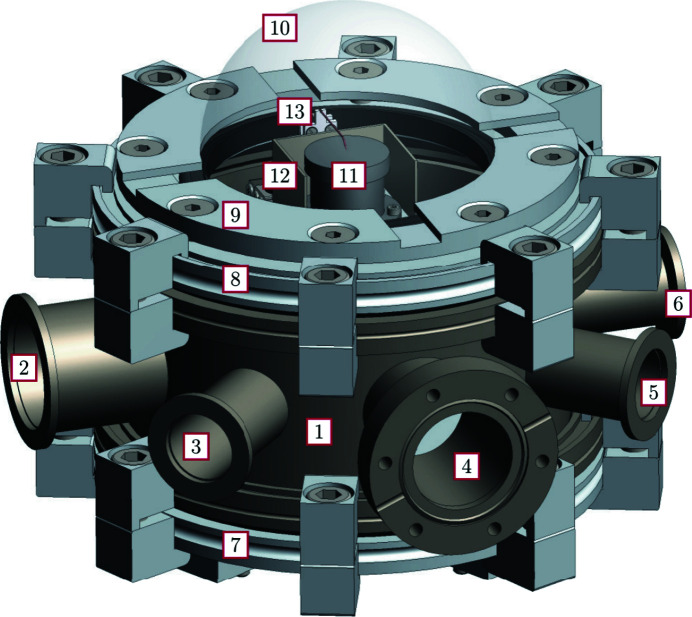
Technical drawing of the environmental chamber. 1: vacuum cross; 2–6: different ports; 7: bottom; 8/9: lid; 10: dome; 11: heater/cooler with inner heat shield; 12: outer heat shield; 13: *xyz* piezo stage.

**Figure 2:**
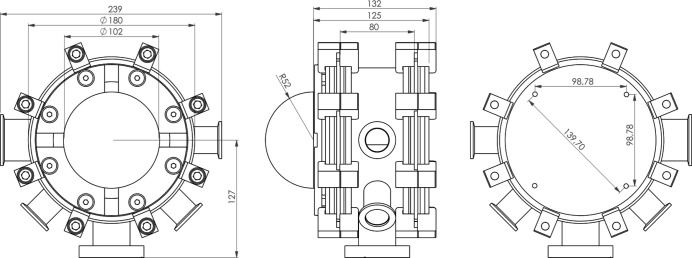
Technical drawing of the top, side and bottom view (left to right) of the environmental chamber with measurements given in mm.

The vacuum chamber (Fig. 1 ▸) weighs in total less than 7 kg at a height of ∼180 mm and a diameter of maximum 239 mm (see Figs. 2 ▸ and 3 ▸). The chamber houses a heating/cooling assembly (11) also representing the sample holder, an outer heat shield (12), and an *xyz* piezostage (13) with probing tip for electrical manipulation. The distance between sample position and bottom of the chamber is 125 mm which equates to the minimum distance required between the diffractometer sample mount and the X-ray beam to accommodate this chamber.

As the chamber will be used for experiments under extreme conditions in terms of electric field and temperature, special care is needed for thermal and electrical insulation. Both points are addressed by restricting the chamber to experiments under high vacuum.

## 3. Vacuum system   

An advantageous basis of the chamber design is a highly modular vacuum vessel. Fig. 1 ▸ gives a detailed overview of the chamber parts, consisting of a standard ISO-K reducing cross (1), of stainless steel 304 with two ISO-K160 ports (lid and bottom), three KF25 ports (3, 5, 6), one CF40 rotatable port (4), and one KF40 port (2) for electrical feedthroughs, the vacuum system, the heater power supply and control, as well as cooling pipes. By replacing the relatively cheap ISO-K reducing cross, arbitrary ports can be implemented to this setup. The bottom is closed with an ISO-K160 blank flange, whereas the lid consists of an ISO-K160 flange with a circular hole (8) (inner diameter of 102 mm), a fixation ring (9), and the dome (10). A vacuum sensor Edwards AIGX-S-NW25 (Edwards, 2020 ▸) is attached to port (3). Other ports will be discussed in the following sections.

**Figure 4:**
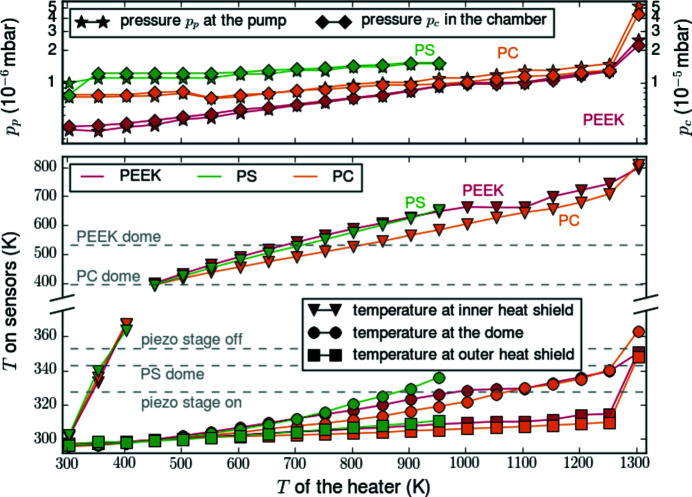
Top: pressure development at the pump *p*_p_ and in the chamber *p*_c_. Bottom: temperature development at the inner heat shield of the heater, at the outer heat shield (12), and at the topmost point on the outside of the dome. The gray dashed lines mark critical temperatures defined by different chamber components, which should not be exceeded at the outer heat shield or at the dome.

We performed heating tests to characterize the chamber’s high-temperature compatibility, which is limited by potential outgassing or melting of the dome materials PEEK, PS and PC (polyether ether ketone, polystyrene and polycarbonate, respectively (Sections 4 and 5). In particular, outgassing leads to increased pressure that, in turn, leads to higher thermal conductivity and therefore even higher dome temperatures. Before each measurement, the vacuum pumps have been left running for at least 24 h to provide highest vacuum conditions. The pressure was measured with vacuum sensors at the chamber and at the pumping station, with respective values *p*_c_ and *p*_p_. Fig. 4 ▸ shows that the initial pressure at *T* = 300 K depends strongly on the dome materials: *p*_c_ = 3.9 × 10^−6^ mbar for PEEK, 7.7 × 10^−6^ mbar for PC, and 1.2 × 10^−5^ mbar for PS (Section 5). With increasing temperature the pressure increases almost linearly. A sudden increase of pressure has been observed for heater temperatures above 1250 K for different dome materials. This may be connected to the critical temperature of the SmarAct stage (13) of 328 K, as explained in Section 4.

## 4. Temperature control   

**Figure 5:**
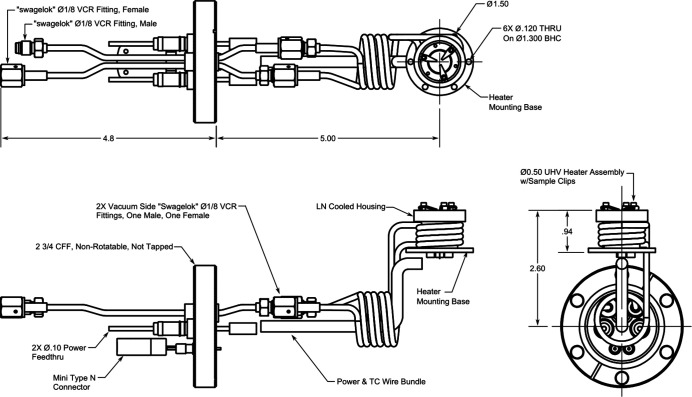
Technical drawing of the heater/cooler assembly with a short description of the components; measures given in inches (HeatWave Labs, 2020*a* ▸,*b* ▸).

The setup for the temperature control consists of a heater/cooler assembly (11), an outer heat shield (12), a feedthrough (4) using the cooling-circuit for water, air, or liquid nitrogen (LN), temperature sensors, and a temperature control unit. The heater/cooler assembly is a customized version of the heater model 104200 by HeatWave Labs Inc. (HeatWave Labs, 2020*a* ▸,*b* ▸). Fig. 5 ▸ shows the setup, which consists of a 0.5′′ ultra-high-vacuum (UHV) button heater with two cylindrical shields. Threaded holes in the inner shield allow fixing a sample with metallic or ceramic clips. Additionally, the heater includes a single type N thermocouple, a LN-cooled heater base, and stress-relieved cryogen tubes, which are fed through a CF40 flange mount at port (4). Beside the cRIO control-system (compact reconfigurable in-/out-modules), the heater may be also controlled by any PID controller such as the available Omega CN8PT-305-006-C24-EIP (Omega Engineering GmbH, 2020*b* ▸). Typical operation settings of the heater are, for example, 7.8 V at 2.4 A for 1550 K and 12.1 V at 3.2 A for 1750 K, according to the supplier (HeatWave Labs, 2020*a* ▸,*b* ▸). A maximum short-term peak power of 60 W is possible, but reduces the heater lifetime.

The type N thermocouple is attached below the heating unit, the cooling components are outside the heater. Therefore, the thermocouple tends to measure slightly too high values at low temperatures.

To protect the equipment inside the chamber from thermal radiation, we attached an additional heat shield (12) (outer shield). Next to the thermocouple of the heater, we added two Pt1000 sensors to the setup (type Omega P1K0-516-8K-B-300-S (Omega Engineering GmbH, 2020*a* ▸) to be applied at several possible mounting positions (*e. g.* at the inner and outer heat shield). These sensors are specified for a temperature range of 73 K to 1123 K. All sensors can be connected to a Fischer connector [S 104A086-130+ (Fischer Connectors, 2020*b* ▸)] within a flange socket at port (6). Currently, the temperature values can be read out by an Omega data acquisition module Pt104A (Omega Engineering GmbH, 2020*c* ▸). Prospectively, the read-out will be automated (Section 7).

The system has successfully been tested up to temperatures of 1300 K. At this point, the temperature at the outer shield (12) rises above the operating limit of the SmarAct stage (13) of 328 K. The lubricant of the SmarAct stage releases gas, which leads to an increase of pressure, which in turn increases the temperature due to the additional heat transfer. The replacement by an UHV SmarAct stage (SmarAct GmBH, 2020*d* ▸), which can be operated at up to 423 K, may enhance the temperature range of the setup. However, to reach 1300 K, the heater was already operated at its long-term stability limit. Therefore, these two facts defined the limit for the feasible temperature range to 1300 K. In the case of using the PS dome, the operation range is limited to a maximum of 1200 K (Fig. 4 ▸) due to the critical temperature of the material.

**Figure 6:**
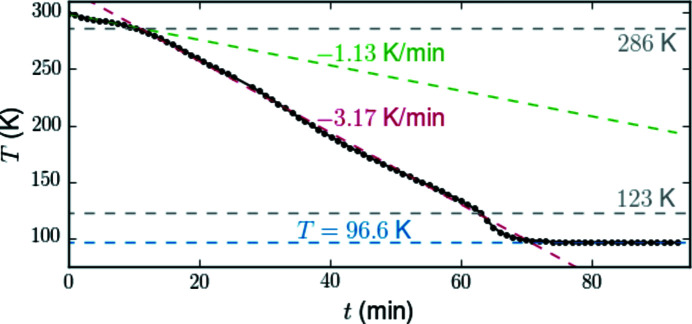
Decrease of the temperature measured at the heater/cooler assembly during cooling with liquid nitrogen with the heater switched off.

Hoses for coolant can be connected directly via VCR 1/4′′ fittings at port (4). The lowest temperature measured at the sample position was at 100 K, which depends on the quality of insulation of the hoses to reduce thermal losses during the LN transport (Fig. 6 ▸). Starting at room temperature, the initial cooling rate was −1.13 K min^−1^ limited by the heat capacity of the hoses. This is followed by an increase to a constant value of −3.17 K min^−1^ for the major part of the cooling range. An intermediate increase of the cooling range just above the low limit may be due to a decreasing heat capacity of the assembly.

## 5. Domes   

**Figure 7:**
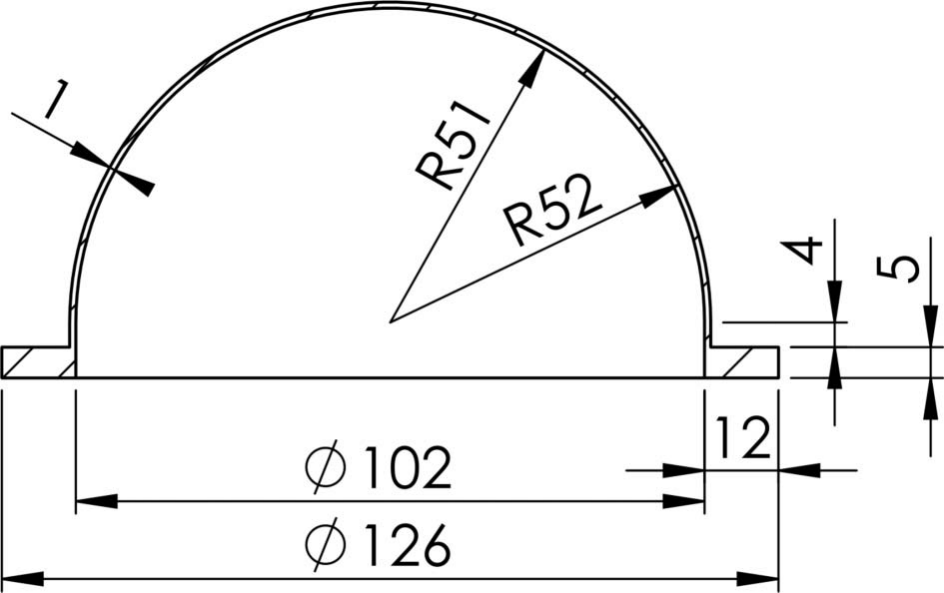
Technical drawing of the PEEK dome with measurements given in mm.

Using X-ray transparent domes (10) allows the analysis of samples within one hemisphere of solid angle with almost constant absorption conditions. The different domes are almost hemispheric (Fig. 7 ▸) and are fixed between flange (8) and the retaining ring (9). The inner diameter is 4′′ (≃ 102 mm) in order to provide sufficient space for electric manipulation of the sample and to reduce the heat load on the dome material.

The domes made of polycarbonate (PC) and polystyrene (PS) are commercially available and were manufactured by deep drawing. Thus, they have a simple and slightly imprecise shape. The polyether ether ketone (PEEK) domes are custom-made by Anton Paar (Anton Paar, 2020*a* ▸) via turning (Fig. 7 ▸), allowing to have the sample holder centered in the hemisphere. This is not the case for the ready-made PC and PS domes. Therefore, a spacer is provided to fix these domes in the correct position.

**Figure 8:**
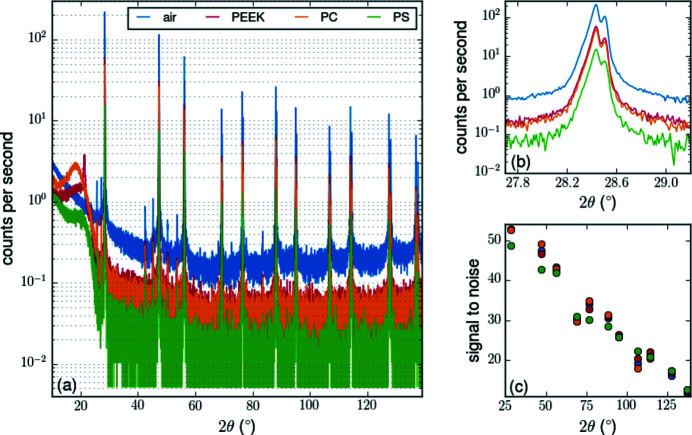
Powder XRD measurements with Cu *K*α radiation of a Si standard sample (NIST SRM 640d) with different domes and without dome (air). The domes were evacuated to a rough vacuum of 280 mbar. (*a*) Complete measured pattern. (*b*) Reflection 111 in detail. (*c*) Signal-to-noise ratio.

We characterized the functionality of the different domes with regard to temperature stability as well as X-ray scattering and attenuation. Fig. 8 ▸(*a*) shows XRD measurements of a standard silicon powder sample (Si NIST SRM 640d powder) with Cu *K*α radiation. The measurements were performed without sample rotation, with symmetric parallel beam (0.1 mm) configuration, 2.5° axial collimation, and a 2.7° 1D detector. During the measurements, the domes were evacuated to a rough vacuum of 280 mbar. Relative to the air measurement, the PEEK dome has the highest average transmission of 25.3%, followed by PC (21.2%) and PS (5.9%). However, the theoretical transmission values in Table 1 ▸ indicate that the PS dome should have comparable experimental values to PEEK. These differences could be due to the actual material parameters being different to those assumed in the calculation that is, for instance, neglecting potential polymer additives. For 2θ angles below 30° using Cu *K*_α_ radiation, the background depends on the scattering geometry and on the dome material [Fig. 8 ▸(*a*)]. Thus, for precise correction the background needs to be investigated before each measurement. At high scattering angles, the signal-to-noise ratio in powder diffraction measurements is not impaired by the presence of the domes, Fig. 8 ▸(*c*).

The maximum working temperature of the heater depends on the dome material corresponding to the different glass transition temperatures, summarized in Table 1 ▸. To assess the operating temperature range for each dome, we attached a type K thermocouple to the top of the domes monitoring its temperature with a handheld data logger during heating. For the PS dome, we aborted the measurement at a heater temperature of 953 K as the glass transition temperature of 340 K was approached on the outside of the dome. This may be due to both a poorer vacuum inside the chamber when using the PS dome as well as higher heat absorption of PS. Using the PC and PEEK domes, the materials’ glass transition temperatures were not limiting the temperature range during the test.

In conclusion, the PEEK dome has the best physical properties for the intended experiments; however, it is also the most expensive choice. The PC dome is the next best choice, offering the additional advantage of being transparent. The usage of the PS dome does not lead to any advantages.

## 6. Electric fields   

The setup for the electrical measurements is based on a customized *xyz* stage for electrical and mechanical micro-manipulation by the SmarAct piezo drive (13) holding a probing needle (right of Fig. 1 ▸). The moving range is 16 mm in the *x* and *y* direction (SmarAct GmBH, 2020*a* ▸) and 12 mm in the vertical *z* direction (SmarAct GmBH, 2020*b* ▸). The position is read out by optical encoders to nanometre-resolution and may be controlled by software or using the hand control module (SmarAct GmBH, 2020*c* ▸). The allowed maximum temperature of the *xyz* stage is 328 K and 353 K during operation and when switched off, respectively. Port (2) provides a LEMO feedthrough (LEMO Elektronik GmbH, 2020 ▸) for two connectors compatible with high-voltage coaxial cables (32 pins, SJG.2B.332.CLASV) controlling the SmarAct stage. For the electrical feedthrough of the sample signal, we used high-voltage feedthroughs [Fischer S 102 A018 ø4.1 (Fischer Connectors, 2020*a* ▸)]. Inside the chamber, one of these two contacts is connected to the probing needle. The second contact may be attached to custom sample carriers, unless a ground connection through the button heater (11) is acceptable. For the measurement of small currents generated for example from pyroelectric characterization, a current amplifier DDPCA-300 from FEMTO Messtechnik GmbH is available (FEMTO Messtechnik, 2020 ▸). High-voltage experiments may employ a Matsusada AMT 5B20 high-voltage amplifier (Matsusada Precision, 2020 ▸), which allows driving samples with up to 5 kV at a slew rate of 360 V µs^−1^.

The chamber itself (*i.e.* the feedthroughs, cables and adapters) is also devised for high-voltage applications up to 5 kV, although the use cases demonstrated here did not require voltages higher than 1000 V.

## 7. Multi-parameter control and monitoring   

The present setup allows to control and monitor temperature and electric field manually and, partly implemented, from beamline P23 of PETRA III at DESY. However, more holistic control and monitoring of all important environmental parameters is desirable and will be achieved by a compact reconfigurable input/output system (cRIO) from National Instruments (NI) (National Instruments, 2020 ▸), which is part of the setup periphery. The components of the system are listed in Appendix *A* [App appa]. The NI cRIO provides real-time control of the setup on account of the included high-speed, high-dynamic-range digital-to-analog, and analog-to-digital converters allowing fast monitoring and control of temperature, electric field *etc*. The system’s programming is based on the NI cRIO-9066 with a dual-core processor and Artix-7 FPGA (field programmable gate array). This high dynamic setup is, among other things, intended for the determination of the pyroelectric coefficient with the Sharp–Garn method (Sharp & Garn, 1982 ▸; Garn & Sharp, 1982 ▸), which we already applied elaborately with a highly precise laboratory setup (Mehner *et al.*, 2017 ▸; Jachalke *et al.*, 2017 ▸, 2018 ▸; Jachalke, 2019 ▸). The therefore already existing software of the laboratory setup is to be adapted for the integration into the synchrotron beamlines P23 and P24 of PETRA III, to allow the synchronization of the synchrotron experiment with the simultaneous external perturbations.

## 8. Examples for applications   

The environment chamber is intended for the extensive investigation of functional materials. The flexible design provides the possibility to customize experiments individually to the given scientific case. In particular, ferroelectrics, requiring simultaneous electric and photon energy dependent characterization, are the target group of the chamber. The following sections briefly describe different examples of applications including structural characterizations of pyro- and ferroelectrics at low temperatures using synchrotron radiation in Section 8.1, structural characterizations of a field stabilized phase over a wide temperature range with synchrotron radiation in Section 8.2, and the electric characterization of a ferroelectric using high electric fields in a small temperature range without structural characterization in Section 8.3.

### 8.1. Synchrotron studies of functional materials at low temperatures   

Even without using the full capabilities of the chamber, highly interesting scientific cases can be investigated. For instance, the low-temperature regime allows the separation of thermal-motion-induced contributions (Ovchinnikova *et al.*, 2005 ▸, 2010 ▸) and point-defect-induced contributions (Dmitrienko & Ovchinnikova, 2003 ▸; Ovchinnikova *et al.*, 2005 ▸) to the reflection intensity. This separation is particularly important while measuring RXD at ‘forbidden’ reflections, which is a powerful technique to study atomic displacements (Richter *et al.*, 2014 ▸, 2018 ▸) and the local electronic structure of selected atomic positions in crystals (Zschornak *et al.*, 2014 ▸).

**Figure 9:**
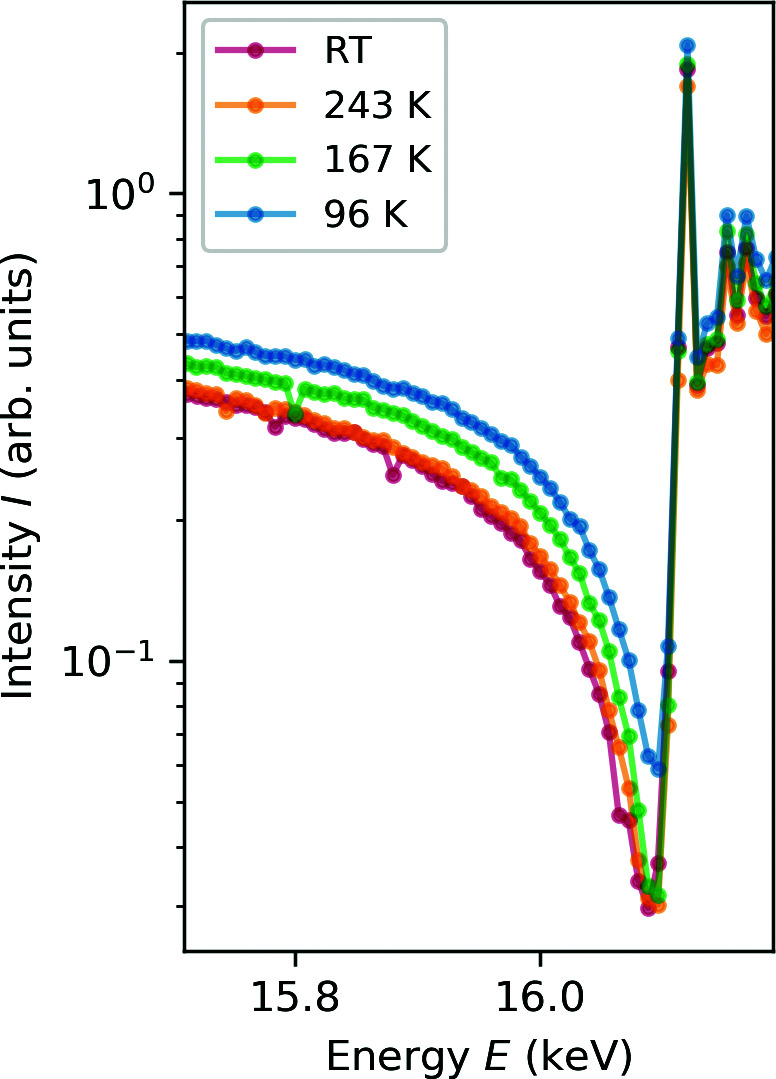
RSD measurement of the thin film sample, exemplarily for the 025 reflection at four different temperatures.

Additionally, the variation of temperature often induces phase transitions, such as for example with strontium titanate (SrTiO_3_), which is an incipient ferroelectric with a low transition temperature (Lytle, 1964 ▸). This ferroelectricity can be stabilized by a variety of external factors such as compressive strain, which allows the investigation of the ferroelectric phase at much higher temperatures. Here, our chamber was used to conduct a resonant XRD experiment at different low temperatures between 96 K and 300 K at nine oblique Bragg reflections to study an expected structural relationship (Nentwich *et al.*, 2021 ▸) to a recently discovered piezo- and pyroelectric phase, the so-called migration-induced field-stabilized polar (MFP) phase (Khanbabaee *et al.*, 2016 ▸; Hanzig *et al.*, 2013 ▸, 2015 ▸; Stöcker *et al.*, 2017 ▸) with the resonantly suppressed diffraction (RSD) approach (Richter *et al.*, 2018 ▸). This new method allows sub-picometre precision by analyzing a purposely induced intensity minimum of an energy-dependent Bragg reflection. These minima originate by compensating scattered intensities of the different atomic partial structures for specific energies close to absorption edges of the constituents. Those minima depend on the structure of the sample. Thus, the shape and position of the intensity minimum of our measurements show a temperature dependence (see Fig. 9 ▸), which could be fitted by a suitable model. Final results will soon be published (Nentwich *et al.*, 2021 ▸).

### 8.2. Synchrotron study of the electroformation of strontium titanate at different temperatures   

The presented sample environmental chamber was recently used for the combined variation of temperature and electric field during the study of structural symmetry reduction in the MFP phase of SrTiO_3_ (Hanzig *et al.*, 2013 ▸, 2015 ▸; Khanbabaee *et al.*, 2016 ▸; Stöcker *et al.*, 2017 ▸). The application of approximately 1 MV m^−1^ causes a structural transition of cubic SrTiO_3_ with space group 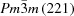 (Hanzig *et al.*, 2013 ▸) to a *P*4*mm* tetragonal structure with elongated *c* direction accompanied by a polar distortion of the TiO_6_ octahedra in the field direction (Richter *et al.*, 2018 ▸). This involves the change of mechanical properties at the cathode side due to oxygen vacancy accumulation (Stöcker *et al.*, 2010 ▸) as well as the appearance of new physical properties in the MFP layer at the anode side such that the SrTiO_3_ crystal becomes partially piezo- (Khanbabaee *et al.*, 2016 ▸) and pyroelectric (Hanzig *et al.*, 2015 ▸). Up to now, the research of the MFP phase is limited to 284 K to 323 K (Hanzig *et al.*, 2016 ▸). Here, we will present first low temperature XRD data of this phase.

**Figure 10:**
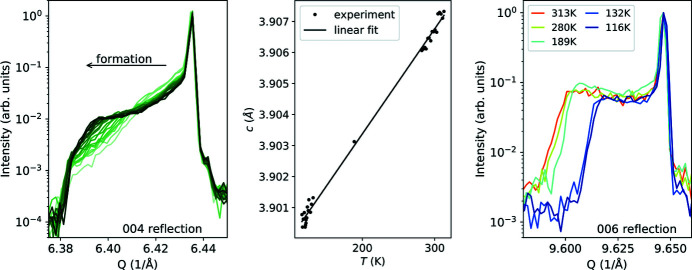
Application of an electric field of 1 MV m^−1^ at different temperatures in the environment chamber to investigate strontium titanate SrTiO_3_ with XRD at photon energy *E* = 16.0 keV. Left: development of a shoulder at lower 2θ values relative to the main peak of the 004 reflection induced by the formation of the migration-induced field-stabilized polar (MFP) phase at 313 K over 8 h (time development: bright to dark). Middle: temperature dependent *c* parameter of cubic SrTiO_3_ derived from the main peak’s maximum intensity of rocking scans. Right: the shoulder formed by the MFP phase at the 006 reflection is more pronounced at elevated compared with low temperatures, which indicates that part of the MFP phase volume relaxes back to the cubic state (*Q* corrected for thermal expansion).

In an experiment at beamline P23 of PETRA III, we extended the temperature range, in which the MFP phase has been investigated so far. We used the presented setup to apply 500 V to a SrTiO_3_ single crystal slab of the size 5 mm × 5 mm × 0.5 mm with platinum electrodes. We applied a temperature of 313 K to the sample, as we observed a faster formation of the MFP phase at slightly elevated temperatures. As we expect structural changes along the *c* direction, we focused on 00*l* reflections. At the chosen energy of ∼16 keV only reflections with *l* ≥ 3 were adjustable from which the strongest are still fairly weak (004 and 006 with 4.3% and 0.6% of maximal intensity, respectively). As the MFP phase is sensitive to photocurrent, the primary beam needs to be attenuated. The left frame of Fig. 10 ▸ shows the evolution of the 004 Bragg reflection during 8 h of formation. The developing shoulder corresponds to the tetragonal MFP phase, having a slightly larger *c* parameter.

Subsequently, we reduced the temperature stepwise and took a rocking scan of the 006 reflection at a time until we reached a temperature of 116 K. The middle frame of Fig. 10 ▸ presents the lattice parameter *c* derived from the main peak’s maximum intensity. Linear regression gives a linear expansion coefficient of 8.65 × 10^−6^ K^−1^ deviating only 0.36% from the value 8.62 × 10^−6^ K^−1^ of Itoh *et al.* (1994 ▸), proving a very accurate temperature control of the chamber. The 0.0048 Å difference (0.12%) in lattice parameter *c* can be assigned to usual uncertainties in the diffractometer setup/a polar splitting of the oxygen and titanium partial structure along the *z* direction. The right-hand frame of Fig. 10 ▸ shows a selection of the underlying rocking scans, which were corrected for the thermal expansion of the *Q* value to highlight the intensity shrinkage of the shoulder with decreasing *T*. It is evident that some volume of the crystal relaxed back from polar to cubic state. The cause of this effect is not yet clear. The experiment shows that the MFP phase remains stable at low temperatures down to 116 K.

### 8.3. Laboratory measurements of the ferroelectric hysteresis of lead zirconium titanate   

To demonstrate the electric characterization capabilities of the setup, we chose lead zirconium titanate PbZr_*x*_Ti_1–*x*_O_3_ (PZT) as a well known model ferroelectric (Yimnirun *et al.*, 2007 ▸; Jachalke, 2019 ▸). Generally, ferroelectric materials offer a wide field of potential applications, ranging from pyro- and piezoelectric sensors, electro-mechanical actuators, and surface acoustic wave devices to non-volatile memory cells (Izyumskaya *et al.*, 2007 ▸; Muralt, 2000 ▸; Moazzami *et al.*, 1992 ▸; Muralt *et al.*, 2005 ▸). For decades, improving the properties of PZT, *e.g.* by minimizing fatigue and imprint, by optimizing polarity and by exploiting exaggerated material properties at the morphotropic phase boundary, has been of scientific and technical interest (Izyumskaya *et al.*, 2007 ▸). In particular, the processing and improvement of thin films was of main concern (Karthik & Martin, 2011 ▸). On the other hand, bulky ceramic PZT is the industry standard of piezoelectric-based actuator technology used for very precise positioning devices.

Here, we demonstrate ferroelectric hysteresis measurements of such a material to characterize the spontaneous polarization of the sample as a function of temperature. The sample was a PIC153 type ceramic from PI Ceramics Germany with a nominal Curie temperature of *T*_C_ ≃ 433 K (PI Ceramics Germany, 2020 ▸). The pre-contacted ceramic has a thickness of 0.26 mm with an area of 19.6 mm^2^; it was fixed with silver conductive paint on the platinum coating (back contact) of a 500 µm-thick sapphire plate. Sapphire and sample did not project beyond the edge of the heater to guarantee a uniform heat propagation. The top contact was realized by the needle held by the SmarAct stage.

The measurements have been conducted at sample temperatures between 323 K and 440 K. 20 minutes after setting a new temperature, the conductivity and the dielectric loss factor tan(δ) were determined with a Hewlett-Packard 4284A LCR bridge at a frequency of 20 Hz with drive amplitude of 1 V to calculate the relative permittivity ɛ_r_. Finally, the *P*–*E* diagrams were recorded with the Shunt method, similarly to Jachalke *et al.* (2018 ▸) and Schenk *et al.* (2014 ▸), by continuously cycling the electric field between −1.6 MV m^−1^ and 1.6 MV m^−1^ at a frequency of 10 Hz and 128 repetitions. Simultaneously, the voltage drop over a reference resistor was measured and converted to the charging current *I* of the sample capacitor (Schenk *et al.*, 2014 ▸). Finally, integration of *I* and division by the contact area *A* yields the polarization *P* = 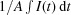. The intercepts of the hysteresis curve with the coordinate axis represent the coercive field *E*_C_ and the remanent polarization *P*_R_, respectively.

**Figure 11:**
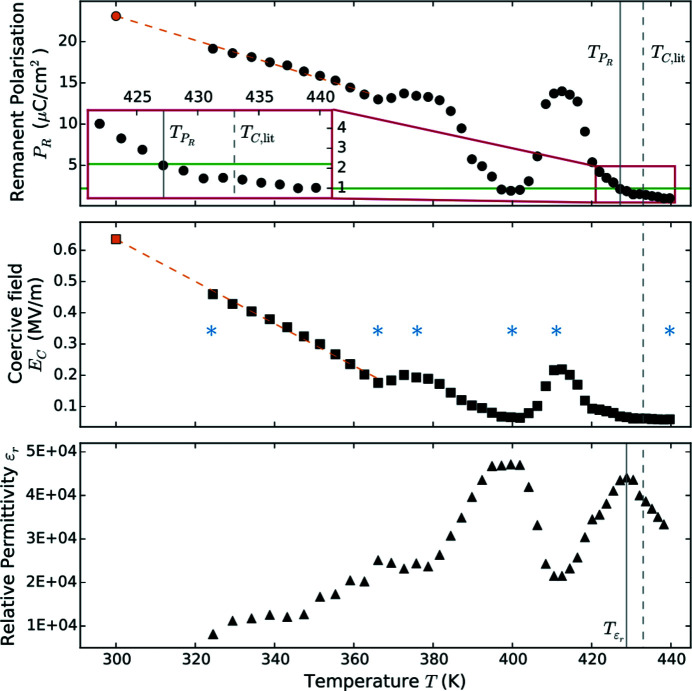
Temperature dependence of remanent polarization *P*_R_, coercive field *E*_C_, and relative permittivity ɛ_r_ of a PIC 153 type PZT sample. On the one hand, the Curie temperature was determined as the local maximum of the relative permittivity at 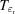 = 429 K, which is lower than the reported value of *T*_C,lit_ ≃ 438 K. On the other hand, the residual signal of the measurement setup was determined for *P*_R_ and the drop below this limit is associated with the phase transition (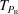 = 427 K). The room temperature values for *P*_R_ and *E*_C_ were extrapolated (orange) as 23.4 µC cm^−2^ and 0.65 MV m^−1^, respectively. Two further phase transitions are visible at ∼375 K and ∼412 K. The temperatures marked with blue asterisks were used for the hysteresis plot in Fig. 12 ▸.

**Figure 12:**
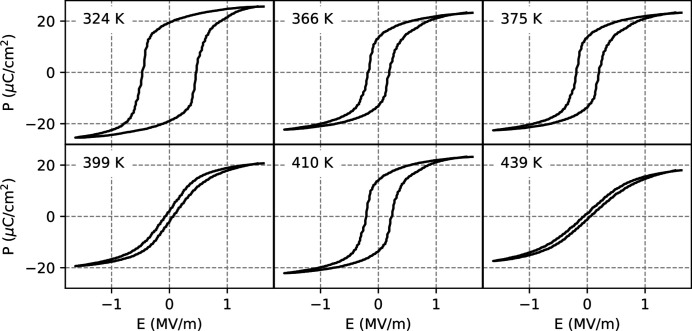
*P*–*E* diagrams showing the hysteresis loops of a PIC 153 type PZT sample at different prominent temperatures: lowest and highest temperatures measured (324 K and 439 K) as well as positions of local extrema in temperature dependence (366 K, 375 K and 399 K). These temperatures are also marked with asterisks in the temperature dependence plot in Fig. 11 ▸.

Fig. 12 shows the hysteresis loops at different prominent temperatures, which are also marked in the *T*-dependence plots of *E*_C_, *P*_R_ and ɛ_r_ in Fig. 11 ▸. Here, we determined the Curie temperature as the local maximum in the relative permittivity ɛ_r_ at 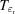 = 429 K. In a second approach, we defined the average error of *P*_R_ as a residual signal, which is undercut in the vicinity of the Curie temperature. We determined the remanent polarization *P*_R_ for repeated measurements under the same conditions. The error of these measurements was calculated as the sum of the standard deviation of *P*_R_ and a relative instrumental error of 11% (Jachalke, 2019 ▸). The instrumental error can be further reduced by a more sophisticated acquisition system attached to the chamber. The average of these errors is ∼2.21 µC cm^−2^ and is undercut for temperatures above 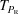 = 427 K. In addition to *T*_C_, we recorded two further phase transitions at ∼375 K and ∼412 K, visible by local maxima in *E*_C_ and *P*_R_ as well as a local minimum in ɛ_r_. The hystereses of these temperatures are also shown in Fig. 12 ▸, besides the ones at *T*_C_, as well as at the selected range limits.

Our measurements are comparable with those performed by Kamel *et al.* on a PZT sample with composition PbZr_0.415_Ti_0.585_O_3_ and crystal size 5 mm × 5 mm × 0.2 mm (Kamel *et al.*, 2007 ▸). They determined a *T*_C_ of 443 K as well as *P*_R_ and *E*_C_ at room temperature of 35.7 µC cm^−2^ and 0.75 MV m^−1^, respectively. Extrapolating the lowest temperature data of the present work (see Fig. 11 ▸), we determined *P*_R_ and *E*_C_ at room temperature of 23.4 µC cm^−2^ and 0.65 MV m^−1^. We assume that our sample has a similar, but not identical stoichiometry as the one used by Kamel *et al.* (2007 ▸), from which our results deviate by 34% and 13% for *P*_R_ and *E*_C_, respectively. Similarly to our measurements, Kamel *et al.* found a residual *P*_R_ above the Curie temperature (Kamel *et al.*, 2007 ▸), which is typical for relaxor ferroelectrics (Safari *et al.*, 1996 ▸).

### 8.4. Laboratory measurements for the electrical characterization of sapphire   

**Figure 13:**
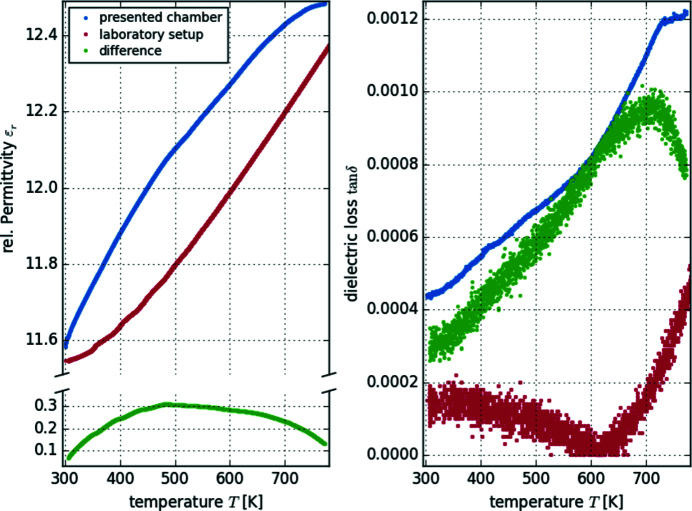
Relative permittivity ɛ_r_ (left) and dielectric loss tan(δ) (right) of a sapphire plate measured with the presented chamber (blue) and with an established laboratory setup (red; difference of both in green).

To demonstrate the suitability of our chamber in terms of electrical measurements, we present the temperature-dependent characterization of the dielectric loss tan(δ) and the relative permittivity ɛ_r,33_ of sapphire. Fig. 13 ▸ shows the corresponding data of an established laboratory setup in comparison with equivalent measurements using the presented chamber in a temperature range of 300 K to 700 K. In both cases a Hewlett-Packard 4275A LCR bridge was used.

The temperature dependence contains the imperfections of both setups. For ɛ_r_ the resulting deviations are still within the specified absolute accuracy of the used LCR bridge. The data of tan(δ) exhibit an additional loss of the setup, which is temperature dependent. However, this influence is smaller than a few 1 × 10^−3^ and thus negligible for most applications.

## 9. Conclusion and outlook   

A new sample environment chamber has been designed and constructed for *in situ* synchrotron experiments allowing the variation of temperature and electric field over a wide range. Different dome materials, like PC, PS and PEEK, give the flexibility for distinguished applications depending on the experiment’s specific demands. Table 2 ▸ gives an evaluation of the dome properties, including further dome materials that are considered for procurement in the near future. Currently, the PEEK dome has the best properties for most experiments, except when a visual control of the interior is desired, in which case the PC dome is preferable. An acquisition of additional domes made of beryllium and polyimide (Kapton^®^) is foreseen. Table 1 ▸ gives an overview of the dome properties that are relevant for the intended applications.

The chamber has already been used to characterize the formation of the MFP phase of single crystalline as well as thin film SrTiO_3_ applying 500 V and varying the temperature in the range of 116 K to 313 K, which showed a stability of this phase at low temperatures. Additionally, ferroelectric hysteresis loops of PZT were presented, below and above the nominal Curie temperature of 433 K with applied electric fields of up to 1.6 MV m^−1^. As a result, the temperature dependencies of *E*_C_, *P*_R_ and ɛ_r_ are given, showing Curie temperatures slightly below the nominal value and two additional phase transitions, which have been unreported until now. Both experiments demonstrate the high range of variable parameters of the environment chamber. Further experiments proved the reliability of the temperature and electric measurements conducted with our chamber.

The high-temperature limit of the sample environment is determined by outgassing of the SmarAct piezo positioner as well as the long-term power limit for operation of the button heater. Removing the SmarAct stage on demand or replacing it by a UHV version would allow the application of temperatures above 1275 K, however at the expense of shortened heater life. To protect the domes from these higher temperatures, improved thermal insulation is necessary. Furthermore, the LN hoses will be replaced by more flexible and double-walled insulated ones, to allow easier movement during diffraction measurements and to reduce thermal losses leading to a lower temperature limit more close to the boiling point of LN. A complete automation and synchronization of the chamber components using the NI cRIO and existing scripts from in-house measurements as well as additional domes made of beryllium and polyimide for enhanced performance parameters are planned in a future project.

The chamber is already available for manual within cooperation for users at beamlines P23 and P24 at PETRA III (DESY). The automatized control and measurement, especially for pyroelectric measurements, will soon be implemented.

## Figures and Tables

**Figure 3:**
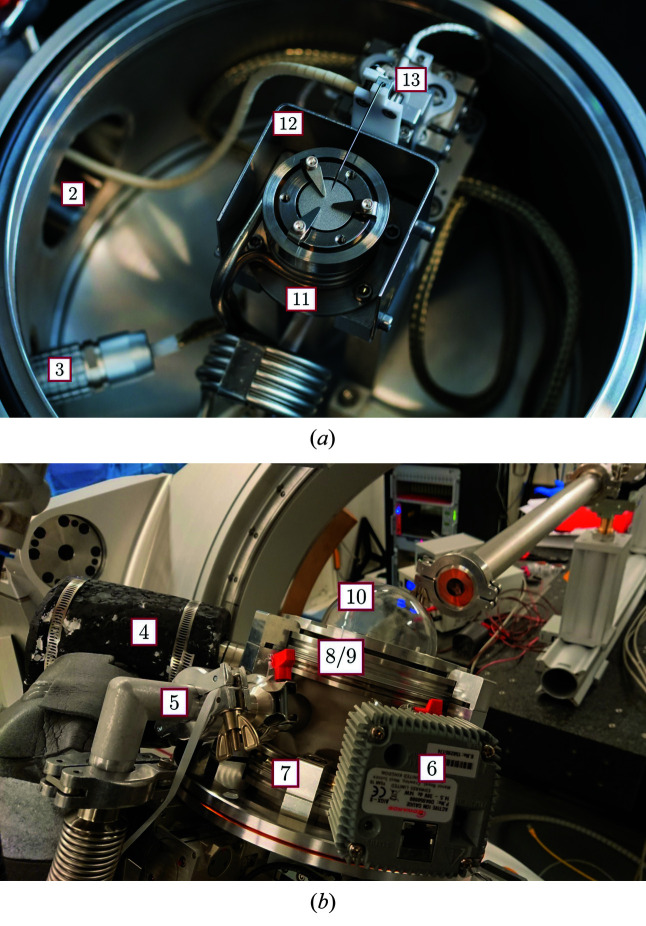
Photographs of the environmental chamber using the same labels as Fig. 1 ▸.

**Table 1:** Properties of the different dome materials (Ensinger, 2020 ▸; Henke *et al.*, 1993 ▸) In addition to the currently available domes made of polyether ether ketone (PEEK), polystyrene (PS) and polycarbonate (PC), we also listed the properties of prospective beryllium (Be) and polyimide (Kapton) domes. The upper limit of the temperature range is determined by the glass transition temperature. Mo *K*α at 17.5 keV and Cu *K*α radiation at 8.0 keV.

	PC	PS	PEEK	Be	Kapton
Color	Transparent	White	Brown/gray	Gray	Orange/brown
Assumed sum formula	C_15_H_16_O_2_	C_8_H_8_	C_19_H_12_O_3_	Be	C_22_O_5_N_2_H_10_
Density (g cm^−3^)	1.20	1.00	1.32	1.85	1.42
Thickness (mm)	1.2	1.6	1.0	0.5	0.25
Price (Euros)	30	30	1800	11000 (+500)	180 (+3500)
Temperature range (K)	213…398	263…343	173…533	…1560	…633
TM† Mo *K*α	0.854	0.861	0.861	0.961	0.957
TM† Cu *K*α	0.232	0.272	0.242	0.827	0.656
AL‡, Mo *K*α (mm)	15.162	21.428	13.331	25.339	11.512
AL‡, Cu *K*α (mm)	1.642	2.455	1.410	5.276	1.187
Radiation stability (kGy)	1000	<1000	20000	∞	40000

† Transmission, double passage through the dome (in and out).

‡ Attenuation length, at 90° glancing angle.

**Table 2:** Evaluation of the dome properties as good (+), average (0) or bad (−), depending on the material (PEEK: polyether ether ketone; PS: polystyrene; PC: polycarbonate; Be: beryllium; Kapton: polyimide)

	PC	PS	PEEK	Be	Kapton
Temperature	0	−	0	+	0
Transmission	0	−	0	+	+
Diffuse scattering	0	+	0		0
Price	+	+	0	−	0
Radiation stability	0	−	+	+	+
Environmentally friendly	+	+	+	−	+

## Appendix A List of available components for the NI cRIO system

The compact reconfigurable input/output system (cRIO) from National Instruments (NI) is intended to control and monitor the external physical fields like temperature and electric field. The whole system comprises the following modules:

(i) Analog input modules for Pt1000 and Pt100 resistance thermometers (NI 9217 and NI 9226).

(ii) A two-channel universal analog input module for reading thermocouples (NI 9218).

(iii) A four-channel analog input module ±10 V, allowing 50000 samples per second at 24 bit resolution (NI 9239).

(iv) A four-channel analog output module ±10 V, allowing 100000 samples per second at 16 bit resolution (NI 9263).

(v) Two digital input/output modules working at TTL voltage levels with frequencies up to 20 MHz (NI 9402 and NI 9403).
